# Smoking and Mortality in Tianjin, China: A Death Registry–Based Case-Control Study, 2010–2014

**DOI:** 10.5888/pcd15.170577

**Published:** 2018-08-16

**Authors:** Wei Li, Guo-hong Jiang, De-zheng Wang, Hui Zhang, Zhong-liang Xu, Ying Zhang, Wen-long Zheng, Xiao-dan Xue, Richard Peto, Tai-Hing Lam

**Affiliations:** 1 Tianjin Centers for Disease Control and Prevention, Tianjin, China; 2 School of Public Health, Tianjin Medical University, Tianjin, China; 3 Clinical Trial Service Unit and Epidemiology Studies Unit, Nuffield Department of Population Health, University of Oxford, Oxford, United Kingdom; 4 School of Public Health, University of Hong Kong, Hong Kong, China

## Abstract

**Introduction:**

We conducted a mortality case-control study to assess the risks of all-cause and major causes of death attributable to smoking in Tianjin from 2010 through 2014. The death registry–based study used data from The Tianjin All Causes of Death Surveillance System, which collects information routinely on smoking of the deceased in the death certificate of Tianjin Centers for Disease Control and Prevention.

**Methods:**

Cases (n = 154,086) and controls (n = 25,476) were deaths at 35 to 79 years from smoking-related and nonsmoking-related causes, respectively. Mortality rate ratios (RRs) for ever smokers versus never smokers, with adjustment for sex, 5-year age group, education, marital status, and year of death, and smoking-attributed fractions were calculated.

**Results:**

The RRs in men were 1.38 (95% confidence interval [CI], 1.33–1.43) for all causes and 3.07 (95% CI, 2.91–3.24) for lung cancer, and in women were 1.46 (95% CI, 1.39–1.54) and 4.07 (95% CI, 3.81–4.35). The smoking-attributed fractions for all causes and for lung cancer in men were 15.4% and 50.2%, respectively, and in women were 7.3% and 32.7%, respectively. Smoking annually caused an average of 3,756 (9.4%) deaths, mostly from lung cancer in men (47.4%) and women (66.9%). Women who started smoking before 30 had a higher RR (1.79; 95% CI, 1.63–1.97) than men who did so (1.48; 95% CI, 1.41–1.56).

**Conclusion:**

Lung cancer was the main cause of smoking-induced deaths in both sexes. Tobacco use is a major cause of premature deaths in men aged 35 to 79 years. Young women must be urged to not start smoking because they could have greater risk of all-cause and lung cancer deaths than men do.

## Introduction

The global epidemic of tobacco-induced deaths needs to be closely monitored in countries at different stages of the tobacco epidemic, especially those with a large number of smokers and a rapidly developing economy like China. Large cohort studies with long follow up are the most commonly used methods but are expensive and need many years of follow up. The most meaningful prospective evidence has come from only a few high-income countries, such as the United Kingdom (1,2) and United States (3), which are at the most advanced stage of the tobacco epidemic (4). Cohort studies are rare in middle- and low-income countries such China, the world’s largest producer and consumer of tobacco with the largest number of tobacco deaths (5,6). One way to estimate tobacco-attributed mortality in developing countries is by mortality case-control (MCC) studies, which study retrospectively a large number of people who have died by assessing their previous smoking habits from interviewing the family and by recording the underlying disease that caused death (7). This method, first initiated by Lam and colleagues (8) and Liu and colleagues (9), should be a much cheaper and faster method to estimate relative risk because it does not need follow up. The first MCC study (9) was done in China by interviewing relatives of the deceased at home at about 1 to 5 years after death, which involved much traveling and labor costs. South Africa was the first country requiring 1 question on smoking on the death certificate, which was the most efficient continuous data collection (10). Two MCC studies followed, in India and Bangladesh, but these were ad hoc community-based studies requiring much research funding (11,12). Hong Kong did a death registry–based case-control study, where relatives were interviewed by research assistants, but the controls used in the analysis were living persons (8).

Tianjin is the third largest city (after Beijing and Shanghai) of China, with an area of 11,920 km^2^ and a population of approximately 12.9 million. The first cigarette factory in China was established in Tianjin in 1891 by Beiyang Tobacco Company, and the industry there controlled more than 70% of the country’s cigarette market (13). Tianjin people were among the first to smoke in large numbers in China. The first China National Smoking Prevalence in 1984 showed that the smoking prevalence in Tianjin was 62.0% in men and 7.0% in women (14). The latter was the highest in China (14). In 2010, the prevalence was 52.9% in men and 2.4% in women, respectively (15). However, relative risks or risk ratios of mortality from smoking in Tianjin have not been reported, and the most recent nationwide cohort study, the China Kadoorie Biobank (CKB), has not included the 3 largest cities (5).

The Tianjin All Causes of Death Registration System (CDRS) was established in 1984, which now covers the entire population of approximately 10 million (as of 2013) with 40% urban and 60% rural residents. The leading causes of death were cardiovascular disease, cancer, and respiratory disease. Through this system, all causes of death should be reported, and all death certificates entered into the database must be completed by physicians in hospitals or community health service centers. Each death certificate has approximately 50 data fields, including age, sex, education, and cause of death, as well as the home address for classifying area of residence as urban or rural. Starting at the end of 2009, Tianjin Centers for Disease Control and Prevention (TJCDC) has been collecting information routinely on smoking (including 3 questions) of the deceased in the death certificate. This is the first and only death registration system in China doing so. Tianjin, being the second to include smoking in death registration in the world after South Africa, collects more information on smoking than South Africa. We used the information routinely collected from the CDRS in 2010 through 2014 to assess tobacco-attributed mortality in the Tianjin population as a whole from particular smoking-related diseases and from all causes, which was the total from all tobacco-attributed deaths from specific causes.

## Methods

### Data collection

Details of the CDRS and some preliminary analysis have been described elsewhere (16). Briefly, the local regulation requires death certification to be completed (including coding the cause of death) and entered into the CDRS computer system by doctors of all hospitals and community health service centers in Tianjin. The public health doctors in district or county CDCs oversee and check the daily reported deaths. Deaths outside hospitals are included in the CDRS based on the information obtained from household interviews and home visits by community clinicians using verbal autopsy as recommended by the World Health Organization (17). The district or county CDCs are responsible for collecting and verifying the medical certificates for all deaths occurring outside of hospitals and adding those deaths to the CDRS weekly, and coding the cause of death according to the *International Classification of Disease, 10th Revision* (ICD-10) (18). The district or county CDCs and TJCDC both carry out data verification, investigation of missing reports, quality control and checking the completeness of the information on the cause and the underlying cause of death codes, and data analysis. TJCDC also provides technical training and support to the staff involved. The proportion of inaccurate coding was less than 3.5% from 2010 through 2014.

Each death certificate has 53 data fields, including age, sex, marital status, education level, home address, and cause of death. Since the end of 2009, there have been 3 questions on smoking on the death certificate: 1) smoking status (answer options are current smoking, quit smoking, and never smoked); 2) number of cigarettes smoked when smoking (answer is the number per day); and 3) total number of years of smoking (answer is the number of years). The information was obtained by doctors through asking relatives living with the deceased. When information was not available, the doctors would leave the answer space blank. During the study period, on average 4.5% of the death certificates had missing data about smoking, and this proportion decreased from 10.9% in 2010 to only 1.6% in 2014. The completeness rates of information on smoking status, number of cigarettes smoked, and total number of years of smoking were 95.5%, 98.6%, and 98.6%, respectively.

Other methods to enhance the quality of the death certification included investigating missing cases regularly in hospitals by random checking of medical records, inpatient registration records, and outpatient and emergency records each year. Investigation of missing cases in the whole population was done through home visits in urban districts and capture-recapture method (matching data from CDRS with Funeral Department records) in rural districts. For deaths in 2010 through 2014, 89.3% of the underlying causes were from the diagnosis by a certifying doctor from a medical institution at or above the county level (36.7% by tertiary [ie, highest level] hospitals, 48.6% by secondary hospitals, and 4.0% by community health service centers). The proportion of unknown causes of death was 1.33%, 1.18%, 1.25%, 1.40%, and 1.60% for each year from 2010 through 2014, respectively.

The accuracy of smoking information was checked through a telephone interview in 2015 on the data from 2010 through 2014 by TJCDC staff. The sample size of the callback survey was calculated according to the proportion of missing information on smoking, reported annually.

The formula of sample size calculation was


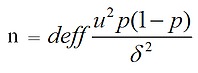


when α = .05, *u* (estimated accuracy rate) = 1.96, *p* = 0.8, δ (tolerance error) = 0.8 × 10% = 0.08, *deff* (design effect) = 1.2, then n = 115. Stratified sampling was performed considering urban and rural area (2 levels), death location (hospital and outside hospital, 2 levels), and smoking status (smoking and not smoking, 2 levels). Taking into account the stratification levels and response rate (about 80%), the total sample size calculation was 115 × 2 × 2 × 2 ÷ 0.8 = 1,150. Compared with the death certificate data, the callback survey had a κ of 0.75, indicating high reliability.

### Design

We included deaths at age 35 to 79 in 2010 through 2014. We excluded deaths at 34 years or younger because smoking was expected to cause very few deaths at a young age, and the number of deaths at 80 or older caused by smoking could be less reliable. We followed the methods of Sitas et al (10), using the same diseases to define cases and controls. Cases were deaths from 11 groups of diseases that are causally or strongly associated with smoking, in 10 groups positively (tuberculosis; chronic obstructive pulmonary disease; other respiratory disease; cerebrovascular diseases; ischemic heart disease; other cardiovascular disease; cancer of the lung; cancer of mouth, pharynx, larynx, or esophagus; cancer of stomach, liver, or pancreas; and cancer of cervix or urinary tract, myeloid leukemia) and in 1 group negatively (Parkinson disease, ulcerative colitis, and endometrial cancer) (10). Controls were deaths from all other specified diseases that were not confirmed to be caused by or were not strongly associated with smoking (Table 1). Similar to Sitas et al, we excluded deaths from Kaposi’s sarcoma, other HIV-related disease, external causes, cirrhosis, or mental and behavioral disorders because any association with smoking could be noncausal. Ill-defined and unknown causes were also excluded. The exposed group included current and former smokers and the unexposed group included never smokers.

**Table 1:** Underlying Causes of Death at Age 35 to 79 Years, Tianjin, China, 2010–2014

Underlying Cause(s) of Death; ICD-10 code(s)	No. (%)
**Cases^a^**	
Tuberculosis; A15–19, B90	360 (0.2)
Chronic obstructive pulmonary disease; I26–28, J40–47	7,774 (5.0)
Other respiratory diseases; J00–22, J30–39, J60–98	5,942 (3.9)
Cerebrovascular diseases; I60–69	45,539 (29.6)
Ischemic heart disease; I20–25, I70–79	46,779 (30.4)
Other cardiovascular disease; I10–15, I30–51, I80–89, I95–99	3,545 (2.3)
Cancer of the lung; C33–34	22,533 (14.6)
Cancer of the mouth, pharynx, larynx, or esophagus; C00–15, C30–32	2,985 (1.9)
Esophagus; C15	1,732 (1.1)
Cancer of the stomach, liver, or pancreas; C16, C22–25	15,713 (10.2)
Stomach; C16	4,503 (2.9)
Liver; C22	7,451 (4.8)
Pancreas; C25	2,783 (1.8)
Cancer of the cervix or urinary tract, myeloid leukemia; C51–3, C55, C64–8, C92	2,425 (1.6)
Cervix; C53	489 (0.3)
Cancer of the endometrium, Parkinsonism, ulcerative colitis; G20, K51, C54	491 (0.3)
**Total cases**	**154,086 (100.0)**
**Controls^a^**	
Diabetes and endocrine; D55–63, D65–89,E03 −34, E65–90	6,507 (25.5)
Infectious and parasitic diseases; A00–14, A20-B19,B25–89,B91–99	308 (1.2)
Other specified cancers; C17–21, C26, C37–40, C42–45, C47–50, C56–63, C69–75, C81–91, C93–96	12,871 (50.5)
Diseases of the nervous system; G00–99, excluding G20	1,458 (5.7)
Digestive diseases; K23, K35–50, K52, K69, K71–73, K75, K77–84, K86–93	889 (3.5)
Genitourinary diseases; N00–99	1,972 (7.7)
Nutritional deficiencies; D50–53, D64, E00–02, E40–46, E50–64	84 (0.3)
Musculoskeletal diseases; M00–99	311 (1.2)
Skin infections and disorders; L00–98	40 (0.2)
Benign neoplasms; D00–48	162 (0.6)
Miscellaneous and minor conditions; O00–99, P00–96, U00–52	9 (0.0)
Acute rheumatic fever or chronic rheumatic heart disease; I00–09	741 (2.9)
Congenital anomalies; Q00–09	119 (0.5)
Oral diseases; K00–14	1 (0.0)
Diseases of the sense organs; H00–62, H68–95	4 (0.0)
**Total controls**	**25,476 (100.0)**

a Definitions of cases and of controls are as in Sitas et al (10).

### Statistical analysis

Unmatched multiple logistic regression was used to calculate odds ratios (OR) or mortality rate ratio of all-cause and cause-specific death in ever smokers versus never smokers adjusted by 5-year age group, education (none, primary, higher, do not know), marital status (never, widowed, divorced, married or living as married, do not know), and year of death (single year from 2010 through 2014).

If D is the total number of deaths (from a particular disease) in smokers, then the number of deaths attributed to smoking, Ds, can be calculated separately for men and women at age 35 to 49, 50 to 64, and 65 to 79 as Ds = D(1 − 1 ÷ OR), where OR is odds ratio (to estimate relative risk), where Ds is positive, Ds divided by the total number deaths in the whole population gives the smoking-attributed fraction (9). Where Ds is negative, the fraction could be due to the “protective” effect of smoking. Attributable numbers for groups of diseases (and for overall mortality) were derived from the sum of the number of each specific disease within the group.

Men and women were analyzed separately. Statistical tests and 2-sided 95% confidence intervals (CIs) were based on changes in log-likelihood. When the 95% CI for the relative risk in men did not overlap with that in women, the sex difference was significant at *P* less than .05 level. Otherwise, the interaction of sex and smoking was tested by adding an interaction term in the model. All analyses were performed with SPSS 16.0 (SPSS Inc).

## Results

From 2010 through 2014 in Tianjin, of all 337,673 deaths registered, 202,249 deaths (59.9%) were persons aged 35 to 79 years, and of these, 187,213 deaths were from the selected diseases. After excluding 7,651 (4.1%) deaths with missing smoking status, we had 179,562 deaths (in 105,531 never smokers, and 74,031 ever smokers, the latter including 55,212 current and 18,819 former smokers) in our analysis on 154,086 cases and 25,476 controls.

### Demographic characteristics and smoking prevalence

Among men, the mean (standard deviation) age at death of cases and controls was 66.3 (10.1) and 64.6 (10.9) years, respectively, and among women was 69.4 (9.0) and 65.6 (10.8) years, respectively. The prevalence of ever smoking was higher in cases in men and women at all age groups (Figure). In men, the prevalence in cases and controls was highest for those aged 50 to 59 years (mean birth year of 1957). In women, the prevalence was lower at lower ages of death. Additional analyses found that the decrease was mainly due to a decrease in uptake of smoking, not an increase in quitting, both in men and in women.

**Figure:**
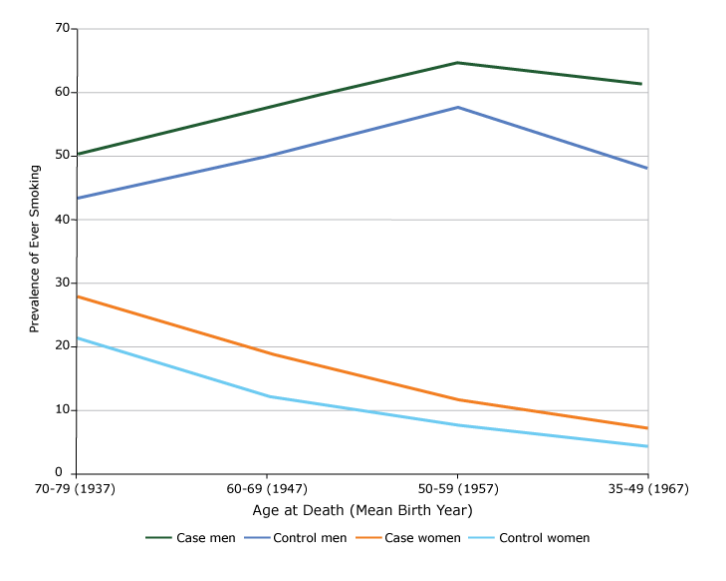
Prevalence of ever smoking by sex and age at death among cases and controls who died at age 35 to 79 years, Tianjin, China, 2010–2014. **Table:** 

Study Group	Age at Death (Mean Birth Year)			
	70–79 (1937)	60–69 (1947)	50–59 (1957)	35–49 (1967)
Case men	50.2	57.8	64.7	61.2
Control men	43.4	50.1	57.7	48.0
Case women	27.8	19.1	11.6	7.0
Control women	21.3	12.2	7.6	4.3

### Rate ratios for selected diseases and deaths attributed to smoking

All-cause rate ratios were significantly elevated in both sexes (Table 2). The rate ratio was significantly higher in women (adjusted rate ratio, 1.46; 95% CI, 1.39–1.54) than in men (1.38; 95% CI, 1.33–1.43) (*P* for sex interaction < .001). The rate ratios were elevated in most of the 11 groups of diseases except the Parkinson disease, ulcerative colitis, and endometrial cancer group with a rate ratio of 0.41 (95% CI, 0.27–0.62) in men. The highest rate ratio was in lung cancer in both sexes, which was also significantly higher in women (4.07; 95% CI, 3.81–4.35) than in men (3.07; 95% CI, 2.91–3.24).

**Table 2:** Mortality Rate Ratio at Age 35 to 79 Years, Selected Causes, Ever Smokers Versus Never Smokers, Tianjin, China, 2010–2014

Cause of Death^a^	Ever Smoker	Never Smoker	Rate Ratio^b^ (95% CI)	No. (%) Attributed to Smoking
**Men**				
Tuberculosis	182	97	1.87 (1.45–2.41)	85 (30.5)
Chronic obstructive pulmonary disease	2,337	1,638	1.67 (1.54–1.80)	938 (23.6)
Other respiratory diseases	1,816	1,864	1.11 (1.03–1.20)	180 (4.9)
Cerebrovascular diseases	14,473	13,727	1.17 (1.12–1.22)	2,103 (7.5)
Ischemic heart disease	14,014	13,440	1.15 (1.10–1.20)	1,828 (6.7)
Other cardiovascular disease	1,357	1,126	1.23 (1.12–1.34)	254 (10.2)
Cancer of the lung	10,117	3,467	3.07 (2.91–3.24)	6,822 (50.2)
Cancer of the mouth, pharynx, larynx, or esophagus	1,493	804	1.89 (1.72–2.07)	703 (30.6)
Esophagus	856	521	1.69 (1.51–1.90)	349 (25.3)
Cancer of the stomach, liver, or pancreas	5,801	4,667	1.25 (1.18–1.32)	1,504 (14.4)
Stomach	1,613	1,442	1.17 (1.08–1.27)	234 (7.7)
Liver	3,069	2,319	1.28 (1.20–1.37)	671 (12.5)
Pancreas	877	674	1.33 (1.19–1.48)	218 (14.1)
Cancer of the cervix or urinary tract, myeloid leukemia	682	548	1.33 (1.18–1.50)	169 (13.7)
Cancer of the endometrium, Parkinsonism, ulcerative colitis	31	86	0.41 (0.27–0.62)	−45
**All causes used as cases (sum of above)**	**52,303**	**41,464**	**1.38 (1.33–1.43)**	**14,402 (15.4)**
**All causes used as controls^c^**	**5,928**	**6,238**	**1 [Reference] **	
**Women**				
Tuberculosis	17	64	1.50 (0.86–2.61)	6 (7.4)
Chronic obstructive pulmonary disease	1,286	2,513	2.17 (1.99–2.38)	693 (18.2)
Other respiratory disease	479	1,783	1.32 (1.18–1.49)	116 (5.1)
Cerebrovascular disease	2,812	14,527	0.92 (0.86–0.98)	−245
Ischemic heart disease	3,951	15,374	1.19 (1.12–1.26)	631 (3.3)
Other cardiovascular disease	196	866	1.27 (1.08–1.51)	42 (4.0)
Cancer of the lung	3,881	5,068	4.07 (3.81–4.35)	2,927 (32.7)
Cancer of the mouth, pharynx, larynx, or esophagus	188	500	1.91 (1.60–2.30)	90 (13.1)
Esophagus	95	260	1.63 (1.27–2.09)	37 (10.4)
Cancer of the stomach, liver, or pancreas	864	4,381	1.17 (1.07–1.28)	126 (2.4)
Stomach	196	1,252	0.96 (0.82–1.13)	−8
Liver	367	1,696	1.24 (1.09–1.41)	71 (3.4)
Pancreas	224	1,008	1.37 (1.17–1.60)	60 (4.9)
Cancer of the cervix or urinary tract, myeloid leukemia	177	1,018	1.23 (1.04–1.46)	33 (2.8)
Cervix	54	435	1.09 (0.81–1.47)	4 (0.8)
Cancer of the endometrium, Parkinsonism, ulcerative colitis	44	330	0.97 (0.70–1.34)	−1
**All causes used as cases (sum of above)**	**13,895**	**46,424**	**1.46 (1.39–1.54)**	**4,378 (7.3)**
**All causes used as controls^c^**	**1,905**	**11,405**	**1 [Reference] **	

Abbreviation: CI, confidence interval.

a Causes of death are causally or strongly associated with smoking, either negatively (Parkinson disease, ulcerative colitis, and endometrial cancer) or positively (all others listed) (10).

b Mortality rate ratio, ever smokers versus never smokers, adjusted for 5-year age group, education, marital status, and year of death.

c All other specified medical causes except cirrhosis and HIV; excluding unknown causes and external causes. See Table 1 for details.

The smoking-attributed fraction of death in men aged 35 to 79 (15.4%; number of deaths, 14,402) was more than 2 times that in women at the same age group (7.3%; number of deaths, 4,378). The 3 leading causes of death attributable to smoking in men were lung cancer (6,822; smoking-attributed fraction: 50.2%), cerebrovascular diseases (2,103; 7.5%), and stomach, liver, or pancreatic cancer (1,504; 14.4%). Among women, the 3 leading causes were lung cancer (2,927; 32.7%), chronic obstructive pulmonary disease (693; 18.2%), and ischemic heart disease (631; 3.3%). Smoking caused 18,780 deaths (9.3% of 202,249 deaths) at ages 35 to 79 years in 2010 through 2014 or on average 3,756 deaths each year, mostly from lung cancer in men (6,822 of 14,402; 47.4%) and in women (2,927 of 4,378; 66.9%).

### Age started smoking

Among men, those who started smoking before age 30 had higher all-cause rate ratios than those who started later for deaths in all age groups, with rate ratios higher in younger age groups (about 1.50 at 35 to 59 and 60 to 69 years) than in the oldest age group (1.38 at 70 to 79 years) (Table 3). For all ages, women who started smoking before 30 had a higher rate ratio (1.79; 95% CI, 1.63–1.97) than men who did so (1.48; 95% CI, 1.41–1.56).

**Table 3:** All-Cause Mortality Rate Ratios, Ever Versus Never Smokers, by Sex, Age, and Age Started Smoking (<30 or ≥30 y), for Diseases Prespecified as Associated With Smoking, Tianjin, China, 2010–2014

Sex/Age at Death	Age Started Smoking <30 Years		Age Started Smoking ≥30 Years	
	No. of Ever Smokers/No. of Never Smokers^a^	Mortality Rate Ratio (95% CI)^b^	No. of Ever Smokers/No. of Never Smokers^a^	Mortality Rate Ratio (95% CI)^b^
**Men**				
35–59	Cases: 10,250/8,881 Controls: 1,384/1,809	1.50 (1.39–1.62)	Cases: 5,172/8,881 Controls: 741/1,809	1.31 (1.19–1.45)
60–69	Cases: 6,167/11,114 Controls: 618/1,653	1.51 (1.37–1.66)	Cases: 8,756/11,114 Controls: 1,003/1,653	1.34 (1.23–1.45)
70–79	Cases: 6,319/21,469 Controls: 582/2,776	1.38 (1.25–1.51)	Cases: 14,919/21,469 Controls: 1,508/2,776	1.28 (1.20–1.37)
35–79	Cases: 22,736/41,464 Controls: 2,584/6,238	1.48 (1.41–1.56)	Cases: 28,847/41,464 Controls: 3,252/6,238	1.31 (1.25–1.37)
**Women**				
35–79	Cases: 4,651/46,424 Controls: 518/11,405	1.79 (1.63–1.97)	Cases: 8,970/46,424 Controls: 1,347/11,405	1.33 (1.25–1.41)

Abbreviation: CI, confidence interval.

a All other specified medical causes except cirrhosis and HIV; excluding unknown causes and external causes. See Table 1 for details.

b Mortality rate ratio, ever smokers versus never smokers, adjusted for 5-year age group, education, marital status, and year of death by unmatched multiple logistic regression model.

## Discussion

This is the first mortality case-control study on smoking and mortality using data routinely collected from death registration in China. Tianjin is one of the largest and most developed cites in China. It has a more sophisticated and reliable system of death registration than most cities in China and other low- and middle-income countries. Through the efforts of many clinical and public health doctors, the quality, quantity, and integrity of death registration all reached China national standards (19). Our test-retest reliability showed good agreement between the smoking data collected by doctors who completed the death registration form and data from telephone interviews by TJCDC staff.

The first mortality case-control study was done by Liu et al in China on deaths in 1986 through 1988 in 98 urban (including Tianjin) and rural areas by interviewing surviving relatives a few years later, in 1989 through 1991 (9). Our rate ratios for all deaths at age 35 to 79 were similar to those at age 35 to 69 in urban areas (1.40 in women and 1.29 in men) in Liu et al (9). However, our smoking-attributed fractions of deaths were higher than those for the whole country in Liu and colleagues’ study and in a nationally representative cohort aged 40 or older at 1991 followed through 1999–2000 in Gu and colleagues’ study in China (6,9), particularly in women (7.3% versus 3% [9] and 3.1% [6] in women, and 15.4% versus 13% [9] and 12.9% [6] in men). Our fraction in men was lower than that in the urban areas from a nationwide cohort study in 2006 (26%) (CKB, which did not include Tianjin) by Chen et al for deaths at age 40 to 79 from 2006 through 2014, but our fraction in women was much higher than theirs (7.3% versus <1% to 5%) (5).

Lung cancer is the disease most strongly associated with smoking and the main cause of smoking-attributed deaths both in men and in women, much more than heart and cerebrovascular and respiratory diseases. The proportions of about half of the smoking-induced deaths attributable to lung cancer in men and two-thirds of deaths in women were probably among the greatest in the world, highlighting that the growing epidemic of tobacco deaths in China have first manifested mainly in increasing lung cancer deaths in Tianjin.

The rate ratios for lung cancer at ages 35 through 79 in our study was 3.07 (95% CI, 2.91–3.24) in men and 4.07 (95% CI, 3.81–4.35) in women. The corresponding rate ratios in the CKB study were 2.98 (95% CI, 2.66–3.33) in urban men and 2.56 (95% CI, 2.02–3.26) in women (urban and rural), were 2.98 in men and 3.24 in women in urban areas in Liu and colleagues’ study, and were 2.44 (95% CI, 2.01–2.96) in men and 2.76 (95% CI, 2.18–3.49) in women in Gu and colleagues’ study. These suggested that while the rate ratios in men are quite similar in the 4 studies (2 MCC and 2 cohort studies) (3,4,7), rate ratios for Tianjin women were the highest. Furthermore, our higher rate ratio for women than men was also consistent with those found in the studies of Liu and colleagues, Chen and colleagues, and Gu and colleagues. Our smoking-attributed fraction of lung cancer in women was also higher than those for the whole country in Liu and colleagues’ study and in the CKB study for urban and rural (32.7% versus 19.4% [9], 9% [5], and 14.8% [6]). But in men, the smoking-attributed fractions were similar in the 4 studies (50.2% versus 52.3% [9], 51% [5], and 50.6% [6]).

Our results suggest that Tianjin women have the highest percentage of all-cause and lung cancer deaths attributable to smoking in China, probably because of their high smoking prevalence. The First National Smoking Prevalence Survey in 1984 showed that the smoking prevalence was 7.04% in women in China, while the prevalence of 27.4% in Tianjin women was the highest (14). However, the annual per capita consumption of cigarettes in Tianjin declined from the peak of 1,064 in 1987 to 728 in 2001 (20). Because there is a gap of several decades between the peak of smoking prevalence and peak of proportion of deaths attributed to smoking (4), the latter needs to be monitored more closely.

If Tianjin is still at an early stage of the tobacco epidemic, the disease burden attributable to smoking in men is expanding in Tianjin despite the decrease in smoking prevalence and per capita consumption, as in China as a whole (5). Because such decreases in China were mainly due to reduction in uptake of smoking rather than increase in smoking cessation in those who stopped by choice for better health (5), our results can forewarn other parts of China and similar countries that much more stringent tobacco control measures are needed to motivate a large proportion of current and relatively healthy smokers to quit by choice.

The rate ratio for all deaths in Tianjin in 2010 through 2014 for ever smokers who started smoking at a young age (<30 years) was 1.79 in women, which was significantly higher than that of 1.48 in men. This rate ratio of nearly 2 in women suggests that the World Health Organization statement that smoking kills 1 out of 2 smokers (21) is also true for Chinese women. As the proportion of smokers who started smoking at young age had increased in the past few decades, and if the increasing rate ratio in the West in the past 5 decades despite decreasing prevalence (22) is repeated in China, the rate ratio is expected to increase rapidly in the next few decades, in men and especially in women. Chen et al predicted that the percentage of deaths attributed to smoking in women could be declining, but they warned that such decline could cease as smoking in adolescent females has increased in some parts of China (5). Although tobacco is a major cause of premature deaths in men aged 35 to 79 years, our results are timely and important to urge young women to not start smoking because they could have greater relative risk of all-cause and lung cancer deaths than men do and have increasing smoking-attributed fractions.

In Tianjin, approximately 70,000 deaths were reported every year during 2010 through 2014, for a crude death rate of 6.5% to 7%. Approximately 85% of the causes of death were diagnosed by secondary hospitals. The validity of relative risk estimates in case-control studies are, to varying degrees, dependent on the prevalence in the control group being representative of the target population. Our control group smoking prevalence was a little lower than for those in the Behavioral Risk Factor Surveillance System (BRFSS) in the Tianjin population (TJCDC, unpublished data, 2010–2012). In our study, any biases of exposure data for controls (including recall bias) should have similarly affected the cases.

The MCC study design using routinely collected smoking data from death certificates can be a quick, efficient, and reliable method to assess and monitor mortality risks of smoking in other cities or countries with a reliable system of death certification and good quality control measures like the Tianjin CDRS. But our study had some limitations. First, the definition of cases and controls would need further research, because more and more diseases have been confirmed to be causally associated with smoking, such as diabetes, which was defined as a control disease in our study following Sitas et al (10). This could have underestimated the relative risk of smoking. Second, our ever smokers included former smokers. As most former smokers stopped smoking because of illness and few stopped by choice in China (5), including them might underestimate the relative risk but would more reliably estimate the smoking-attributed fraction and number of deaths. Questions on whether former smokers stopped by choice should be asked (5) in future case-control and cohort studies, together with duration of stopping. Third, we did not analyze urban and rural data separately. With rapid urbanization of rural areas surrounding big cities like Tianjin, the effects of smoking in urban and rural areas would become similar.

Our findings highlight the high risks of deaths, mainly from lung cancer, in men and women ever smokers and particularly the high proportion of deaths attributable to smoking in women in Tianjin, which had the highest smoking prevalence among women around the 1980s in China. Strong tobacco control measures are needed to motivate a large proportion of smokers, including female smokers, to stop smoking. Special and urgent warnings and tobacco control campaigns are needed to prevent the increase in smoking in young women. In countries, regions, or cities with a reliable system of death certification, the mortality case-control study design using routinely collected smoking data from death certificates can be used to rapidly and periodically assess the mortality risks of smoking and evaluate the effects of tobacco control measures at different stages of the tobacco use epidemic.

This Tianjin study is China’s first mortality case-control study based on smoking data from death registration. Lung cancer was the main cause (half in men and two-thirds in women) of smoking-induced deaths. The smoking-attributed fractions of all-cause and lung cancer deaths in women were the greatest probably because of the high smoking prevalence among woman in the city around the 1980s. The mortality case-control study design can be used to rapidly and periodically assess the mortality risks of smoking and evaluate the effects of tobacco control measures.
